# Supplementary material for: Loss of essential outer membrane functions causes drug hypersensitization in Acinetobacter baumannii overexpressing multidrug efflux pumps
Source: mBio. 2026 Jun 24;17(8):e00902-26. doi: 10.1128/mbio.00902-26 (PMC13463702; doi:10.1128/mbio.00902-26)
Supplement: Supplementary file 1 [file mbio.00902-26-s0008.pdf]

Supplementary Figure Legends

Supplementary Figure 1: Mapping of sgRNAs shows a normal distribution of reads. (A-C) Histograms of the mean reads for all sgRNAs (A) from the original pDNA library prior to introduction into *A. baumannii*; (B) after introduction in the *A. baumannii* ATCC 17978UN (WT) strain background at T1 prior to dCas9 induction (n=3); and (C) all shuffle sgRNAs in the WT strain background at T2 after dCas9 induction (50ng/mL aTc, n=3). Reads were normalized to total mapped reads and log transformed. Reads are binned by increments of 10. (D) Growth rate (doubling times in min) ± SEM (n≥5) for cultures grown in LB broth without drug or inducer, with 100ng/mL aTc inducer alone, with CIP alone (0.1 µg/mL for WT or 60 µg/mL for GPL), or with aTc and CIP both present. aTc: anhydrotetracycline. CIP: Ciprofloxacin. GPL: *gyrA parC adeL*. WT: wildtype. NC_159: non-targeting shuffle control sgRNA.

Supplementary Figure 2: Lack of relationship between target location and fitness. (A-C) Statistical significance (n=10) plotted as a function of fitness differences of hypomorphs in the presence and absence of dCas9 induction (100ng/mL aTc) in the WT strain background. Gray: all sgRNAs. Black: sgRNAs overlapping start codon (A). sgRNAs that from either the 3’ or 5’ end of the sgRNA are 1-10nt (B) or 25-40nt (C) away from the first nt of the start codon. (B, C) Red: sgRNAs downstream the start codon. Blue: sgRNAs upstream the start codon. Dotted line: FDR or Q=5%. (D) Distribution of fitness differences (bin of 0.1) vs. fraction of perfect sgRNAs from each category falling in each fitness range. (E) Expression efficiency of four targets achieved by perfect sgRNAs that showed minimal effects on fitness are depicted alongside that of *gyrA* that shows a strong fitness defect. Efficiency is noted as the percent fold change in

transcript levels observed by hypomorphs that were incubated with dCas9 inducer against those grown in parallel without inducer.

Supplementary Figure 3: Outgrowth of banks for 14-16 generations enhances fitness defects. (A, B) Statistical significance (n=5) plotted as a function of fitness scoring differences after growth for 7-8 generations compared to 14-16 generations in the presence (Black) or absence (Gray) of dCas9 induction (100ng/mL aTc) in WT (A) or GPS (B) strain backgrounds. (C-F) Statistical significance is plotted as a function of fitness changes after growth for 7-8 generations (C, E) or 14-16 generations (D, F) of all hypomorphs upon dCas9 induction. Black: perfect guides. Dark gray: statistically significant sgRNAs passing the FDR or Q=5% via multiple t-test analyses. Light gray: sgRNAs not statistically significant. Dotted red box to highlight shifts in fitness that are observed between conditions. aTc: anhydrotetracycline. WT: Wildtype. GPS: *gyrA parC adeS*.

Supplementary Figure 4: Outgrowth of banks for 14-16 generations does not substantially affect fitness of the shuffle guides. (A-D) Statistical significance (n=5) plotted as a function of fitness differences after growth for 7-8 generations (A, C) compared to 14-16 generations (B, D) of all hypomorphs upon dCas9 induction. Black: perfect guides. Dark gray: statistically significant sgRNAs passing the FDR or Q=5% via multiple t-test analyses. Light gray: sgRNAs not statistically significant. Red: shuffle sgRNAs. Dotted blue box to visualize shifts in fitness observed between conditions. WT: Wildtype. GPS: *gyrA parC adeS*.

Supplementary Figure 5: Depletion of a limited number of essential genes results in RND pump overproducer-dependent fitness defects in the absence of drug pressure. (A-C) Statistical significance plotted as a function of the difference in fitness scores for hypomorphs in GPS (A), GPN (B), or GPL (C) vs. WT in the absence of drug after growth for 7-8 generations (n=10). Of the statistically significant sgRNAs passing the FDR or Q=5% via multiple t-test analyses (Gray), the noted targets show significant fitness score differences at least 20% from WT (limited to targets that are represented by 3 or more sgRNAs in this category, with reads of each sgRNA being at least 10 at T1, excluding ribosomal proteins). Black: sgRNAs not statistically significant. Dotted lines: selection parameters wherein substantial changes in fitness are imparted by sgRNAs. (D, E) Fitness in each strain background generated by sgRNAs of the indicated targets. Targets were identified as in A-C, however, the targets displayed fitness score differences of at least 10% from WT in all three FQR strains (D) or in two FQR strains (E). Dotted line marks neutral fitness value of 1. Untx: untreated. WT: Wildtype. GPS: *gyrA parC adeS*. GPL: *gyrA parC adeL*. GPN: *gyrA parC adeN*.

Supplementary Figure 6: Continued depletion through 14-16 generations in the absence of drug reveals a smaller set of fitness determinants tied to AdeAB overexpression than that identified after 7-8 generations. (A-B) Scatter plots of fitness resulting from indicated sgRNAs. Targets were identified after comparing fitness of hypomorphs in WT (black circles) vs. GPS (blue squares) after 7-8 generations (A, n=10) or 14-16 generations (B, n=5). The respective sgRNAs showed significant fitness score differences of at least 10% between strains, passing the FDR or Q=5% via multiple t-test analyses. Targets had to be represented by 3 or more sgRNAs, reads of each sgRNA at T1 were at least 10, and with ribosomal proteins excluded from analysis.

Dotted line marks a neutral fitness value of 1. Red boxes highlight targets whose sgRNAs in GPS yielded fitness scores that differed by at least 20% from WT. WT: Wildtype. GPS: *gyrA parC adeS*.

Supplementary Figure 7: Identification of sgRNAs that alter fitness of FQR strains relative to WT in presence of CIP. (A) Statistical significance plotted as a function of fitness score differences between hypomorphs grown in CIP and Untx conditions for 7-8 generations in the WT strain background (n=10). Gray: Statistically significant sgRNAs passing the FDR or Q=5% via multiple t-test analyses. Black circles: sgRNAs not statistically significant. 16/18 of the top targets identified as causing significantly altered fitness in the FQR strains compared to the WT strain under CIP stress are highlighted as described for Figure 3, but here in the WT strain background to depict the abated dependence on these proteins for fitness in WT. Dotted lines: selection parameters wherein substantial changes in fitness are imparted by sgRNAs. (B-C) Scatter plots exhibit the mean fitness generated by individual sgRNAs (n=10) corresponding to the 18 top FQR fitness determinants as seen in the WT (black), GPS (gray), GPN (red), or GPL (blue) strains after CIP treatment. These sgRNAs lower fitness of more than one FQR strain in the presence of CIP (B) or provide more than one FQR strain with a fitness advantage during CIP exposure (C). All unique sgRNAs are marked by circles, while the individual sgRNAs causing fitness modifications that varied significantly between FQR and WT strains (and between CIP vs Untx conditions) are labeled by empty squares. The dotted line marks the neutral fitness value of 1. CIP: ciprofloxacin treated (50-65 μg/mL for the FQR strains or 0.11-0.12 μg/mL for WT). Untx: untreated. WT: wildtype. GPL: *gyrA parC adeL*. GPN: *gyrA parC adeN*. GPS: *gyrA parC adeS*.

Supplementary Figure 8: Fluctuations in hypomorph growth rates explained by varied levels of transcript depletion. (A) Mean ± SEM (n=3) growth rate differences (in min) between hypomorphs and a shuffle non-targeting control sgRNA (NC_159). Doubling time differences in untreated and CIP-treated conditions are superimposed. Multiple unpaired t tests were applied with Welch correction and following the two-stage set-up of Benjamini, Krieger, and Yekutieli with a false discovery rate of 5% (33). Target depletions yielding significant differences in doubling times between GPL and WT during CIP exposure are shown: ***, p=0.006793; ****, p<0.0001. (B) Mean fold change ($2^{\Delta\Delta CT}$) ± SEM (n=3) in transcript levels for each target as compared to a non-targeting shuffle sgRNA control (NC_159) in the WT (light gray) and GPL (dark gray) strain backgrounds. Arrows (A, B) point to the unique sgRNAs yielding unanticipated fitness results. (C) Mean fold change ($2^{-\Delta\Delta CT}$) ± SEM (n=3) in transcript levels of *atpF* in GPL during depletion using sgRNA AtpF_20 compared to those observed using a non-targeting shuffle sgRNA (NC_1). The value inside the bar: mean transcript level ($2^{-\Delta\Delta CT}$) relative to the shuffle control; value outside the bar: knockdown efficiency ($2^{\Delta\Delta CT}$) compared to shuffle control. (B, C) Values are negative to display lowered transcript levels resulting from the targeted depletions. CIP: ciprofloxacin (50 µg/mL for GPL and 0.09 µg/mL for WT). Untx: untreated. WT: wildtype. GPL: *gyrA parC adeL*.

Supplementary Figure 9: Variability during LpxC and LptC depletion. (A) Mean ± SEM (n=9) growth rate differences (in min) between hypomorphs and a shuffle non-targeting control sgRNA (NC_159). Multiple unpaired t tests were applied with Welch correction and following the two-stage set-up of Benjamini, Krieger, and Yekutieli with a false discovery rate of 5% (33).

Target depletions yielding significant differences in doubling times between GPL and WT during CIP exposure are shown: ****, p=0.000071. (B) Mean fold change ($2^{\Delta\Delta CT}$) ± SEM (n=9) in transcript levels for each target as compared to a non-targeting shuffle sgRNA control (NC_159) in the WT (black border) and GPL (gray border) strain backgrounds. Transcript levels were evaluated via qRT-PCR in samples harvested from cultures grown in the absence of drug. Values are negative to portray lowered transcript levels resulting from the targeted depletions, and the mean values are noted above the x-axis. (A-B) Color of each data point represents a biological replicate to better relate repression and growth rate results. CIP: ciprofloxacin (50 µg/mL for GPL and 0.09 µg/mL for WT). Untx: untreated. WT: wildtype. GPL: *gyrA parC adeL*.

Supplementary Figure 10: Depletion of IhfB, PhaF, and AtpF in the parental GP and CIP susceptible WT strains. (A, B) Mean fold change ($2^{-\Delta\Delta CT}$) ± SEM (n=3) in transcript levels of each target in GPL + NC as compared to GP + NC (A) or in GPL and GP during IhfB depletion (using *ihfB*_P sgRNA) relative to NC (B). (C, D) Mean ± SEM (n≥8) pHi values are displayed. Hypomorphs were incubated in LB with dCas9 inducer (100ng/mL aTc) to mid-exponential phase (Untx, C) or to early-logarithmic growth then treated with CIP (60 µg/mL for GPL and 0.1 µg/mL for WT) for 1 hour (CIP, D). Cells were then harvested and loaded with BCECF-AM dye for cytosolic pH determination. Ordinary two-way Anova with Dunnett's multiple comparisons test: *, adj. p<0.05. ****, adj. p<0.0001. Grey dotted line with "^2": two datapoints were unable to be properly interpolated from the standard curve but contain 490/440 ratios suggestive of pHi values around 7.5 or higher (C). NC: NC_159, non-targeting shuffle sgRNA. Untx: untreated. CIP: Ciprofloxacin treated. GP: *gyrA parC*. GPL: *gyrA parC adeL*.

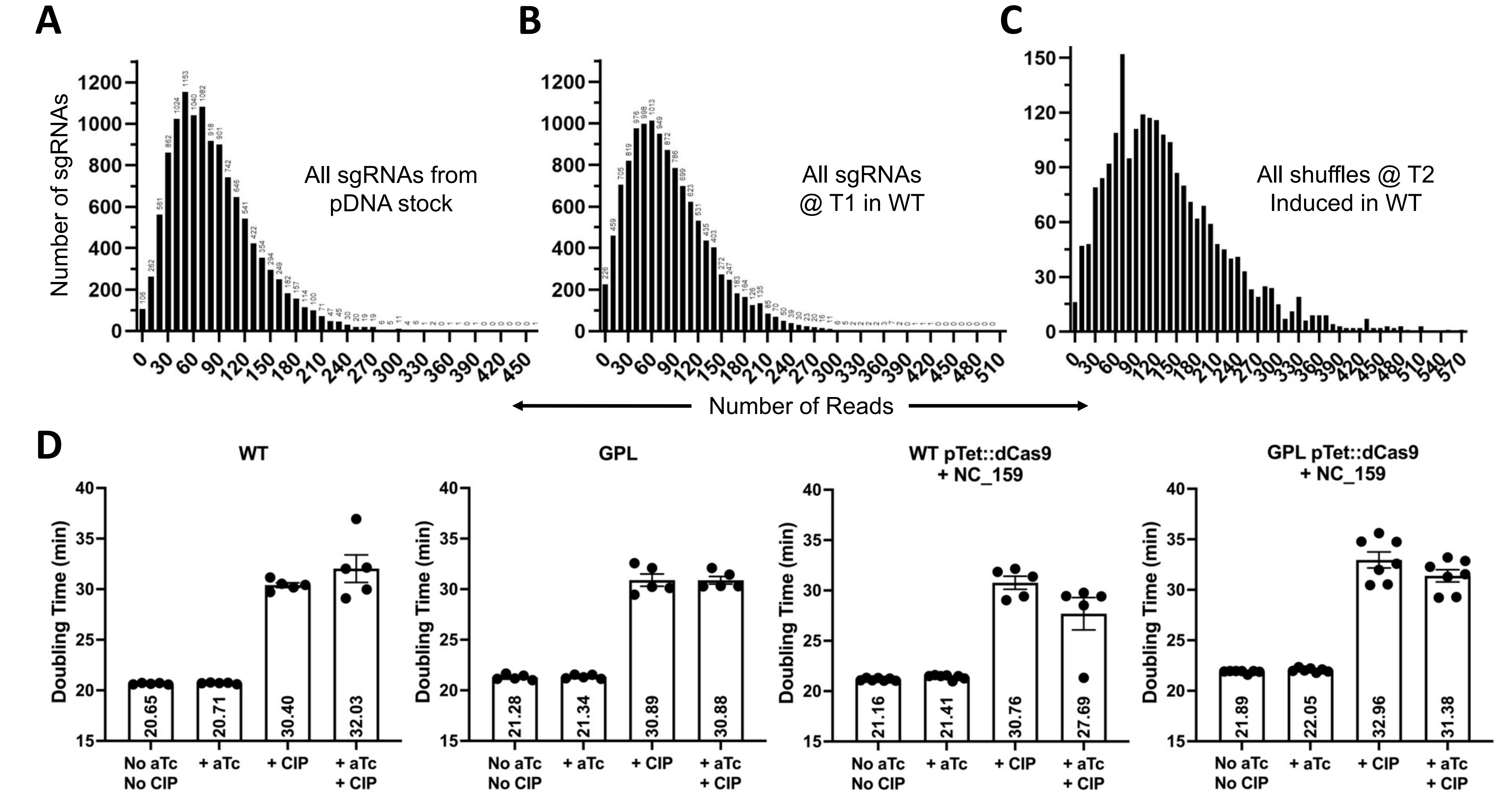

A
B
C
Number of sgRNAs
All sgRNAs from pDNA stock
106
262
561
862
1024
1153
1040
1082
918
901
742
646
541
422
354
294
249
182
157
114
100
71
47
45
30
20
19
19
All sgRNAs @ T1 in WT
226
459
705
819
976
998
1013
949
872
786
699
623
531
435
403
272
247
183
164
126
135
85
70
50
39
All shuffles @ T2 Induced in WT
Number of Reads
D
WT
GPL
WT pTet::dCas9 + NC_159
GPL pTet::dCas9 + NC_159
Doubling Time (min)
20.65
20.71
30.40
32.03
21.28
21.34
30.89
30.88
21.16
21.41
30.76
27.69
21.89
22.05
32.96
31.38
No aTc No CIP
+ aTc
+ CIP
+ aTc + CIP

Supp Fig 1

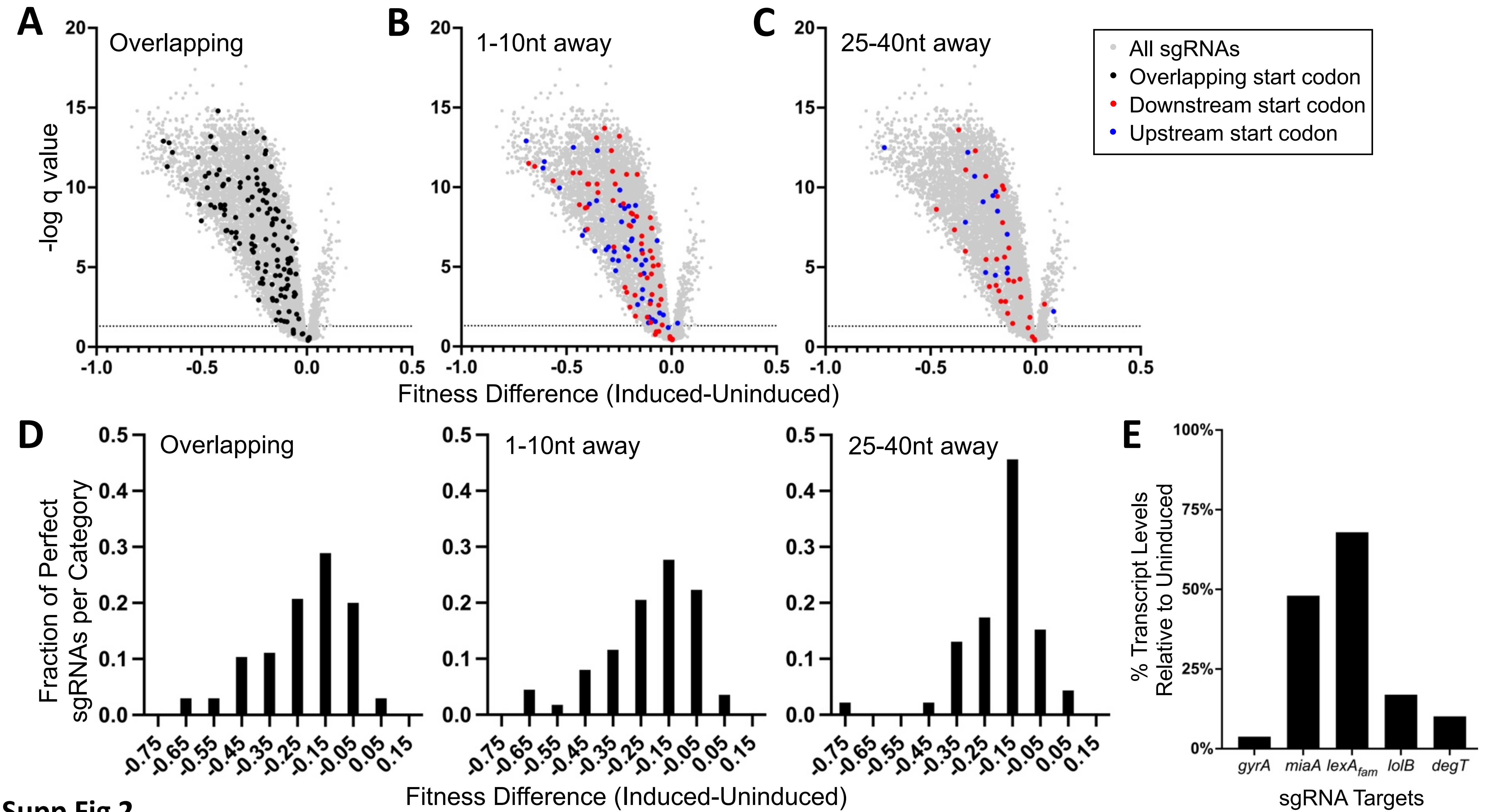
A
Overlapping
B
1-10nt away
C
25-40nt away
All sgRNAs
Overlapping start codon
Downstream start codon
Upstream start codon
-log q value
Fitness Difference (Induced-Uninduced)
D
Overlapping
1-10nt away
25-40nt away
Fraction of Perfect sgRNAs per Category
Fitness Difference (Induced-Uninduced)
E
% Transcript Levels Relative to Uninduced
gyrA
miaA
lexA fam
lolB
degT
sgRNA Targets

Supp Fig 2

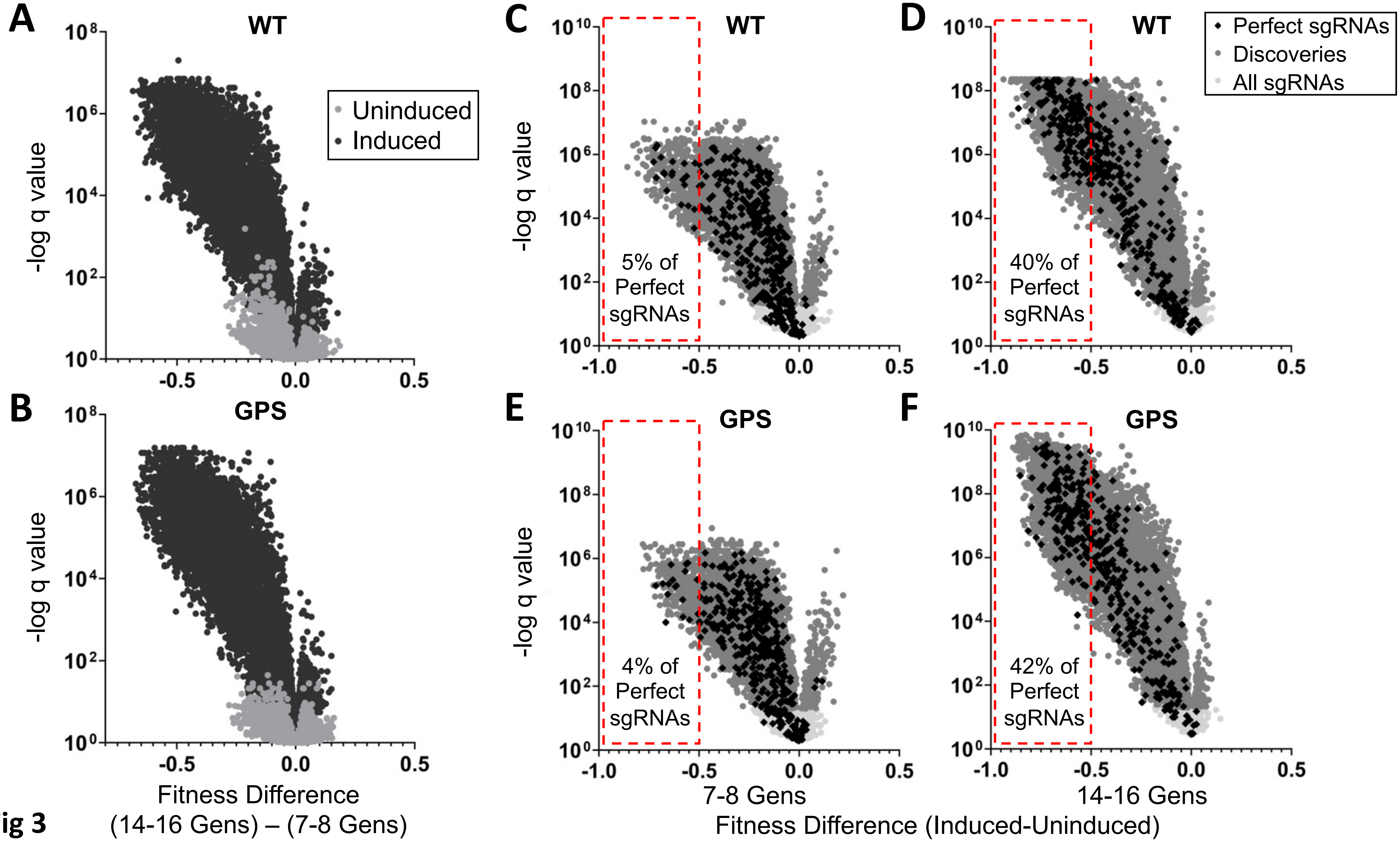
A
WT
Uninduced
Induced
-log q value
B
GPS
-log q value
Fitness Difference
(14-16 Gens) – (7-8 Gens)
C
WT
5% of
Perfect
sgRNAs
-log q value
D
WT
40% of
Perfect
sgRNAs
Perfect sgRNAs
Discoveries
All sgRNAs
E
GPS
4% of
Perfect
sgRNAs
-log q value
F
GPS
42% of
Perfect
sgRNAs
7-8 Gens
14-16 Gens
Fitness Difference (Induced-Uninduced)

Supp Fig 3

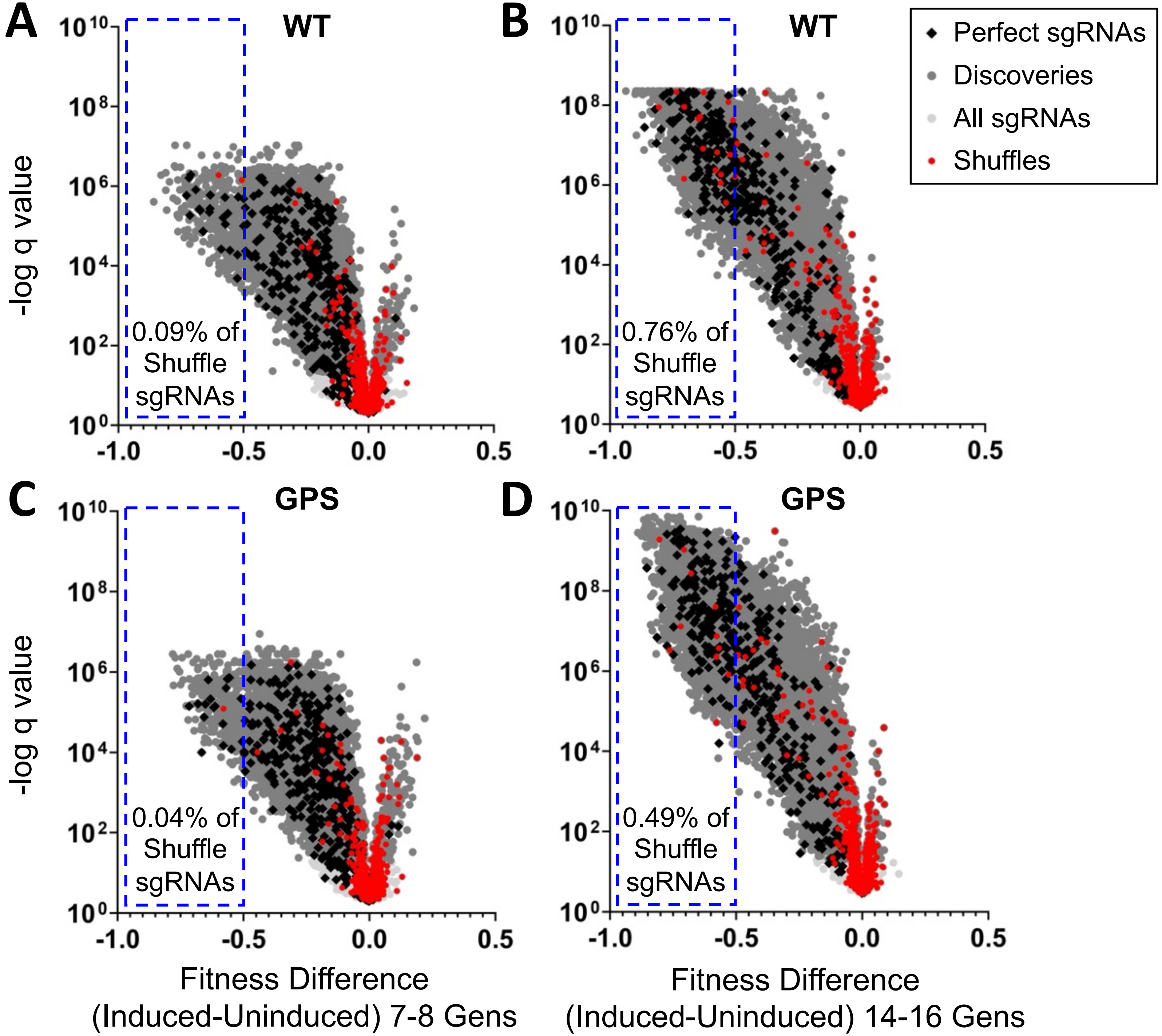
A
WT
B
WT
Perfect sgRNAs
Discoveries
All sgRNAs
Shuffles
-log q value
0.09% of Shuffle sgRNAs
0.76% of Shuffle sgRNAs
C
GPS
D
GPS
0.04% of Shuffle sgRNAs
0.49% of Shuffle sgRNAs
Fitness Difference (Induced-Uninduced) 7-8 Gens
Fitness Difference (Induced-Uninduced) 14-16 Gens

Supp Fig 4

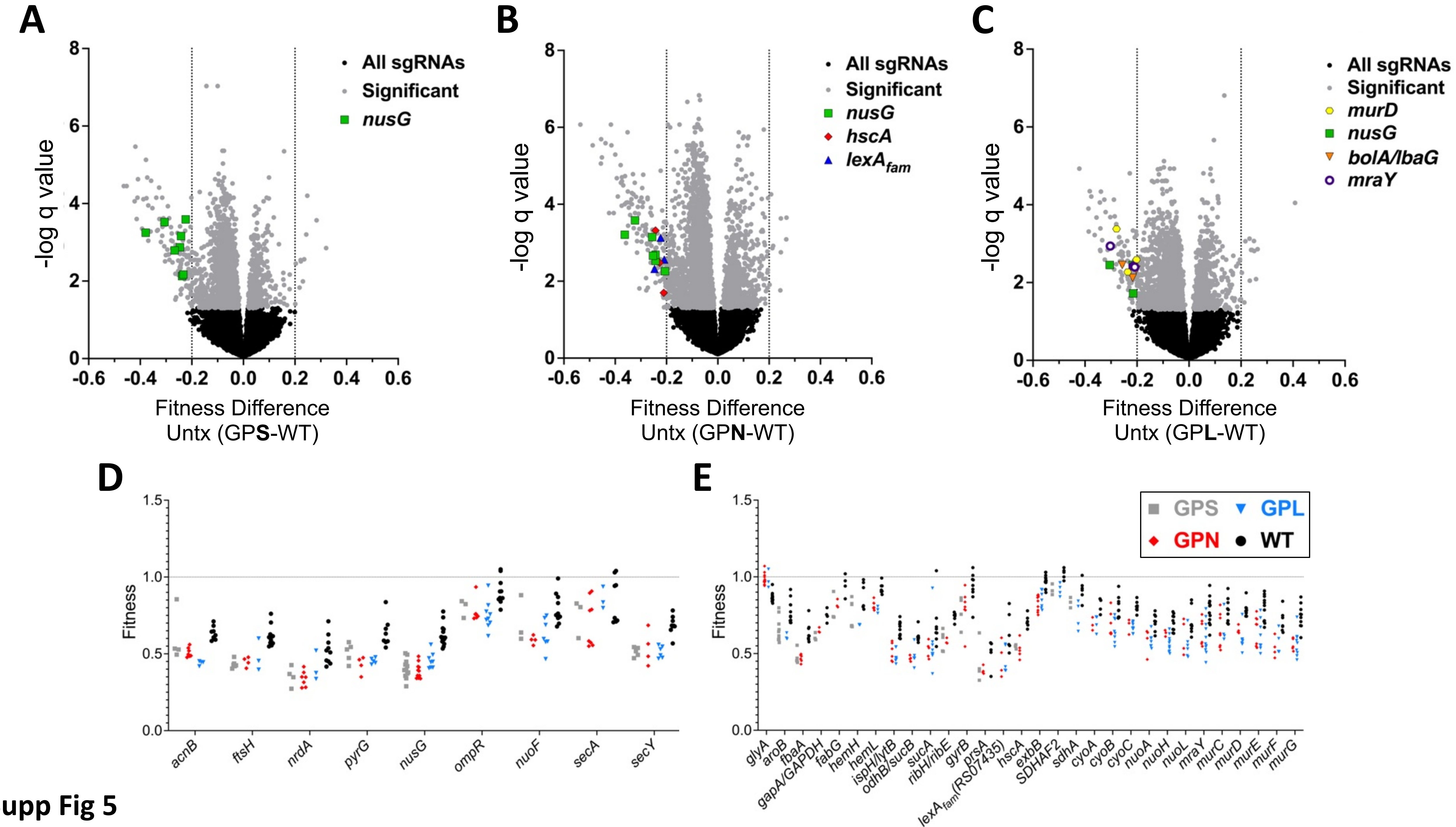
A
B
C
-log q value
Fitness Difference
Untx (GPS-WT)
Untx (GPN-WT)
Untx (GPL-WT)
All sgRNAs
Significant
nusG
hscA
lexA fam
murD
bolA/lbaG
mraY
D
E
Fitness
GPS
GPL
GPN
WT
acnB
ftsH
nrdA
pyrG
nusG
ompR
nuoF
secA
secY
glyA
aroB
fbaA
gapA/GAPDH
fabG
hemH
hemL
ispH/lytB
odhB/sucB
sucA
ribH/ribE
gyrB
prsA
lexA fam (RS07435)
hscA
exbB
SDHAF2
sdhA
cyoA
cyoB
cyoC
nuoA
nuoH
nuoL
mraY
murC
murD
murE
murF
murG

Supp Fig 5

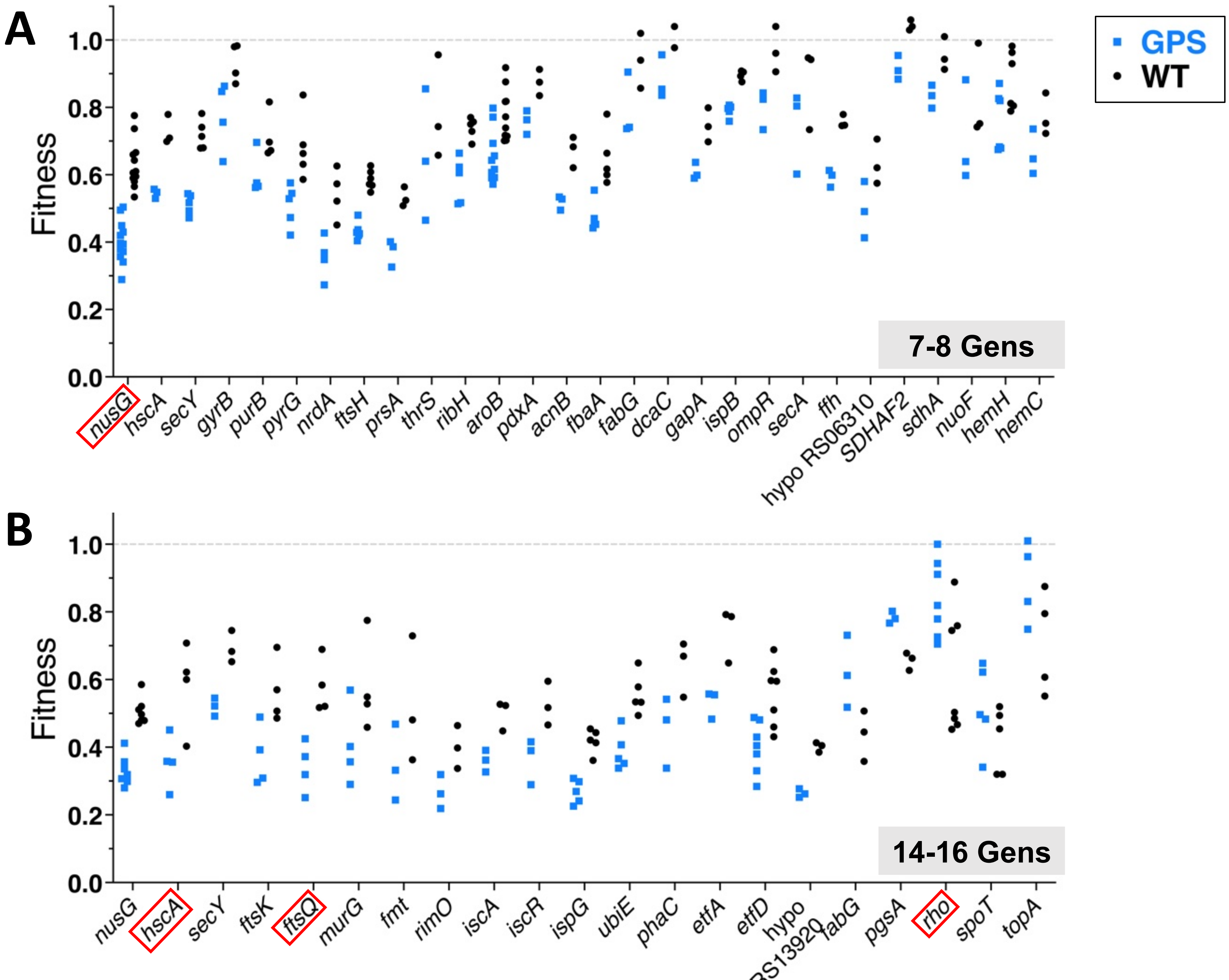
A
B
GPS
WT
Fitness
1.0
0.8
0.6
0.4
0.2
0.0
7-8 Gens
nusG
hscA
secY
gyrB
purB
pyrG
nrdA
ftsH
prsA
thrS
ribH
aroB
pdxA
acnB
fbaA
fabG
dcaC
gapA
ispB
ompR
secA
ffh
hypo RS06310
SDHAF2
sdhA
nuoF
hemH
hemC
14-16 Gens
nusG
hscA
secY
ftsK
ftsQ
murG
fmt
rimO
iscA
iscR
ispG
ubiE
phaC
etfA
etfD
hypo RS13920
fabG
pgsA
rho
spoT
topA

Supp Fig 6

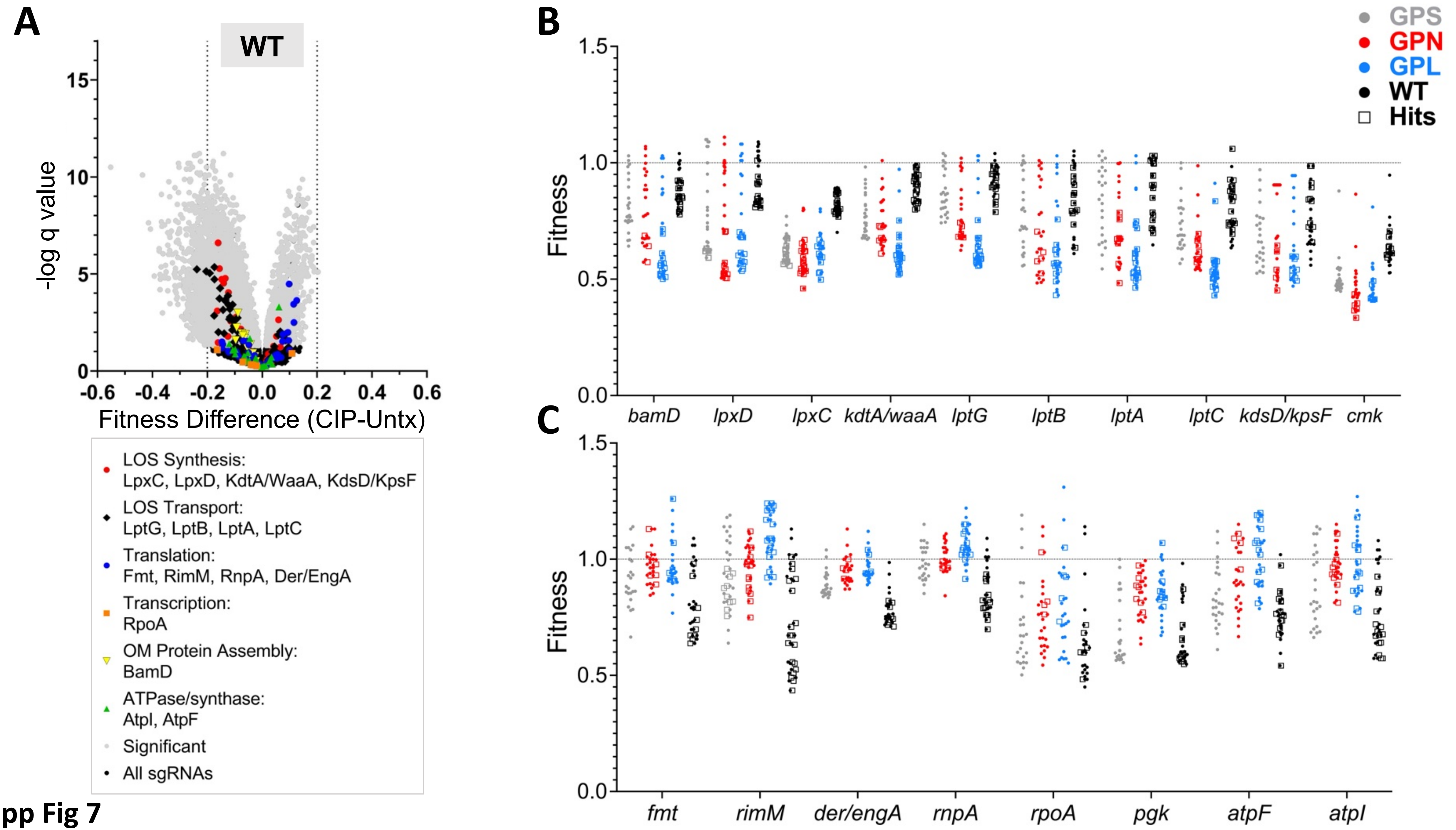

A
WT
-log q value
Fitness Difference (CIP-Untx)
LOS Synthesis:
LpxC, LpxD, KdtA/WaaA, KdsD/KpsF
LOS Transport:
LptG, LptB, LptA, LptC
Translation:
Fmt, RimM, RnpA, Der/EngA
Transcription:
RpoA
OM Protein Assembly:
BamD
ATPase/synthase:
AtpI, AtpF
Significant
All sgRNAs
B
GPS
GPN
GPL
WT
Hits
Fitness
bamD
lpxD
lpxC
kdtA/waaA
lptG
lptB
lptA
lptC
kdsD/kpsF
cmk
C
Fitness
fmt
rimM
der/engA
rnpA
rpoA
pgk
atpF
atpI

Supp Fig 7

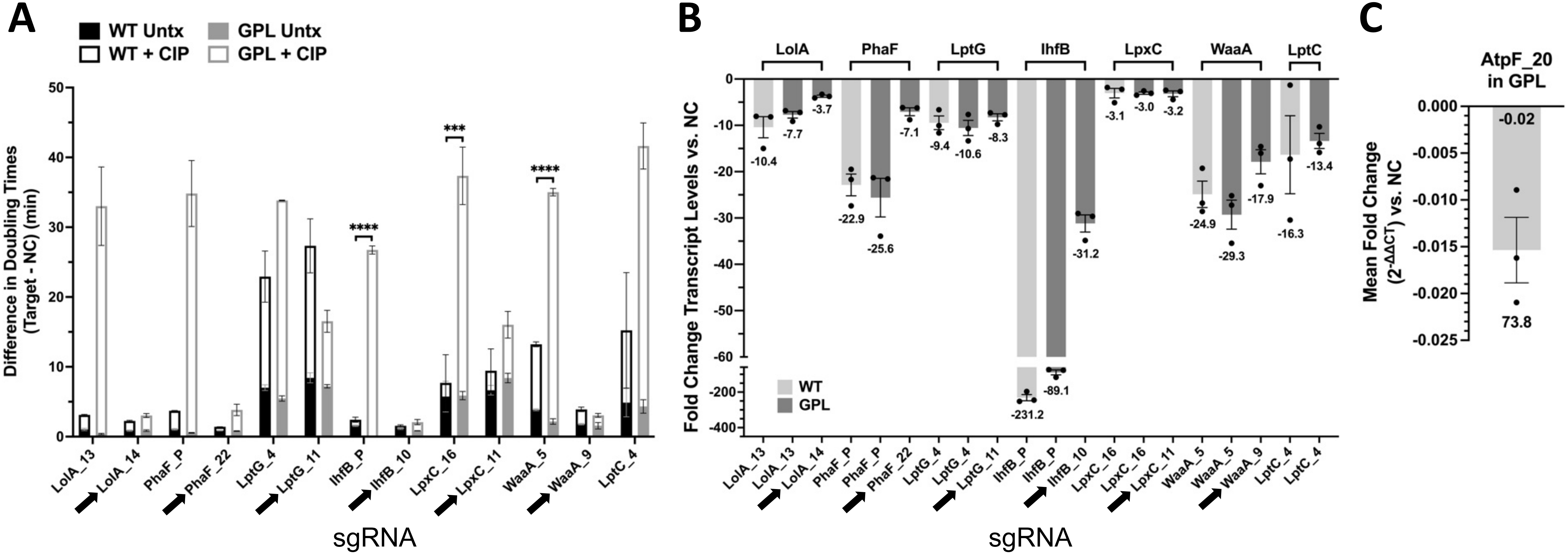
A
WT Untx
GPL Untx
WT + CIP
GPL + CIP
Difference in Doubling Times (Target - NC) (min)
****
***
****
LolA_13
LolA_14
PhaF_P
PhaF_22
LptG_4
LptG_11
IhfB_P
IhfB_10
LpxC_16
LpxC_11
WaaA_5
WaaA_9
LptC_4
sgRNA
B
LolA
PhaF
LptG
IhfB
LpxC
WaaA
LptC
Fold Change Transcript Levels vs. NC
-10.4
-7.7
-3.7
-22.9
-25.6
-7.1
-9.4
-10.6
-8.3
-231.2
-89.1
-31.2
-3.1
-3.0
-3.2
-24.9
-29.3
-17.9
-16.3
-13.4
WT
GPL
sgRNA
C
AtpF_20 in GPL
Mean Fold Change ($2^{-\Delta\Delta CT}$) vs. NC
-0.02
73.8

Supp Fig 8

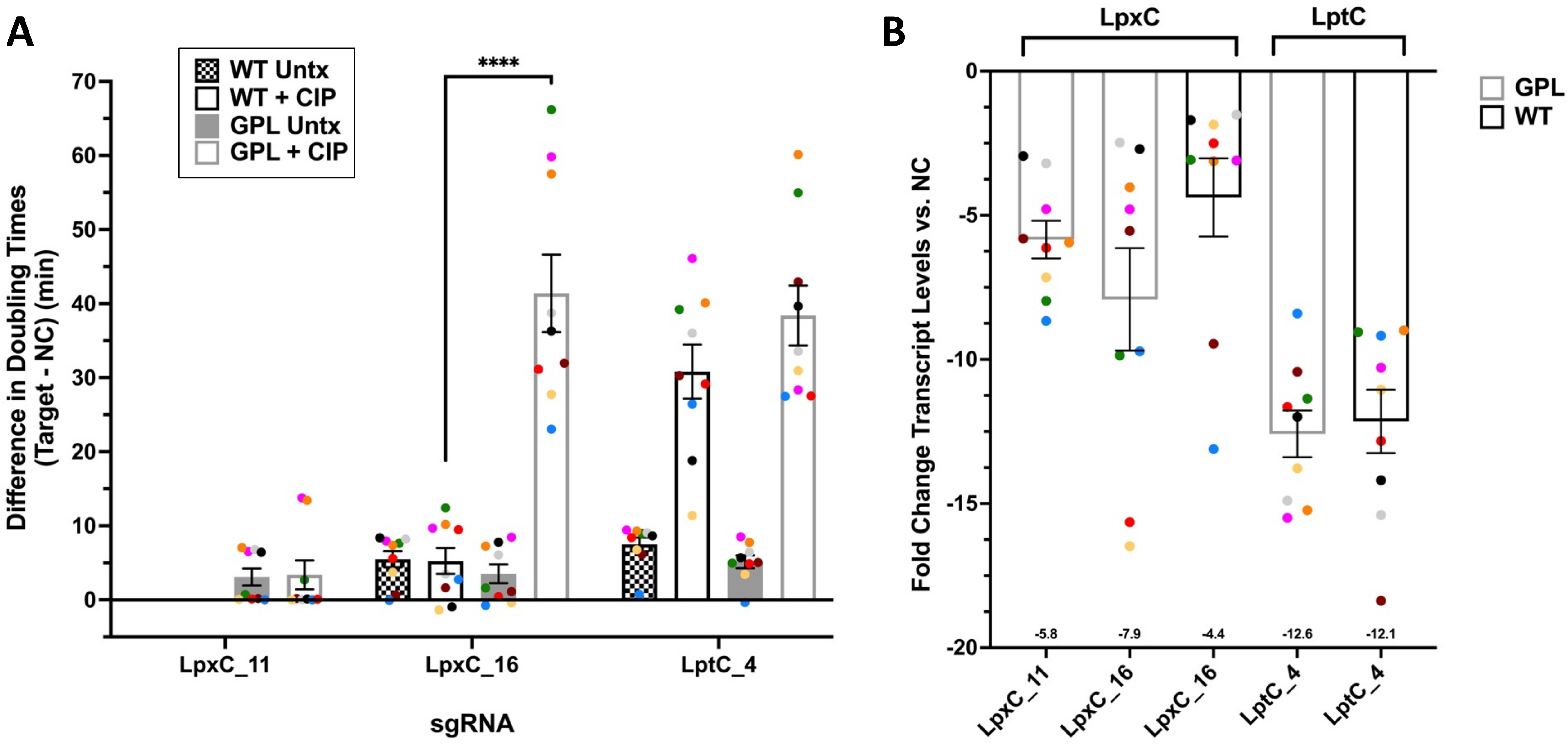
A
WT Untx
WT + CIP
GPL Untx
GPL + CIP
****
Difference in Doubling Times (Target - NC) (min)
70
60
50
40
30
20
10
0
LpxC_11
LpxC_16
LptC_4
sgRNA
B
LpxC
LptC
GPL
WT
Fold Change Transcript Levels vs. NC
0
-5
-10
-15
-20
-5.8
-7.9
-4.4
-12.6
-12.1
LpxC_11
LpxC_16
LpxC_16
LptC_4
LptC_4

Supp Fig 9

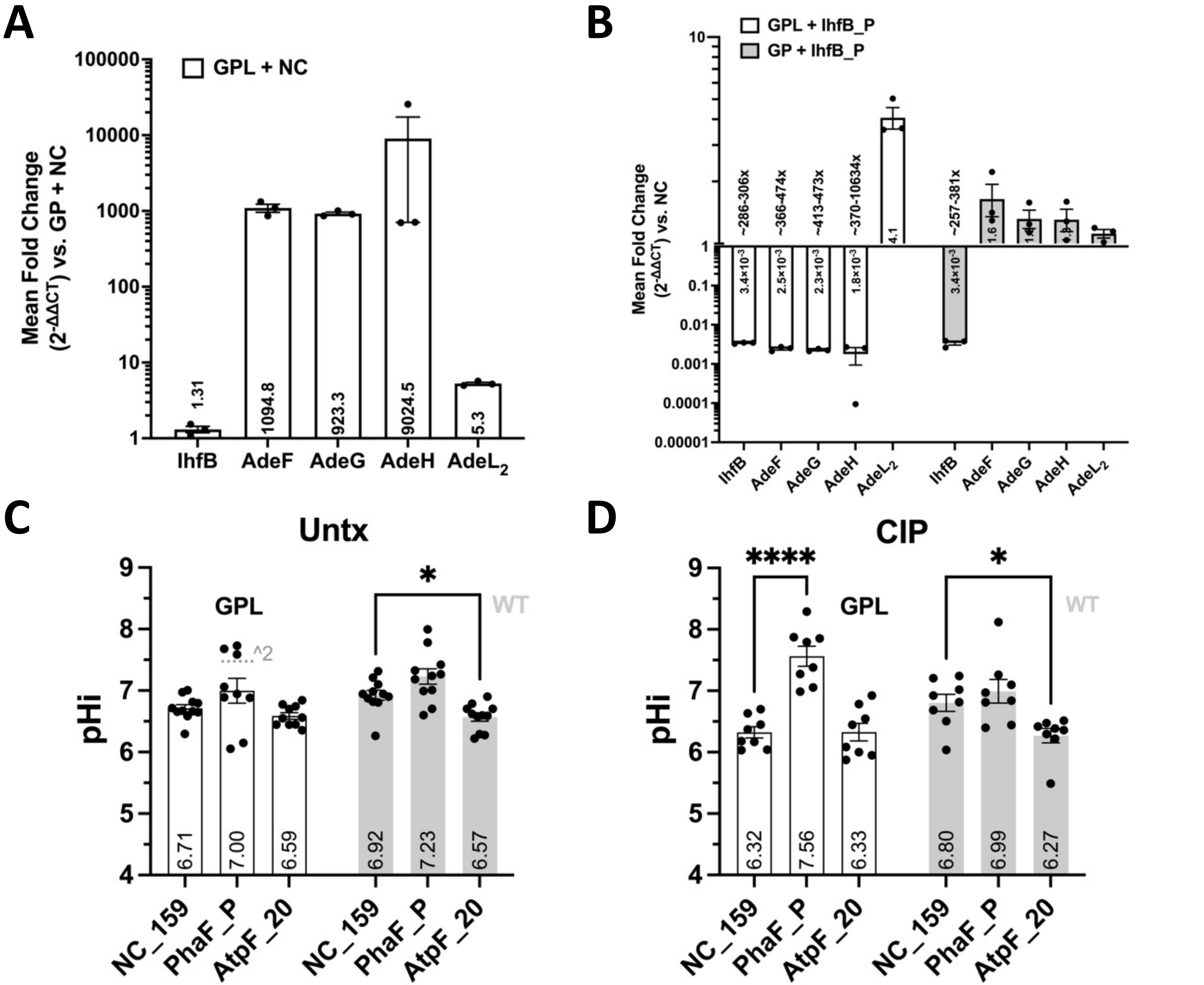
A
GPL + NC
Mean Fold Change (2^-ΔΔCT) vs. GP + NC
1.31
1094.8
923.3
9024.5
5.3
IhfB
AdeF
AdeG
AdeH
AdeL2
B
GPL + IhfB_P
GP + IhfB_P
Mean Fold Change (2^-ΔΔCT) vs. NC
~286-306x
~366-474x
~413-473x
~370-10634x
~257-381x
3.4×10^-3
2.5×10^-3
2.3×10^-3
1.8×10^-3
4.1
1.6
C
Untx
GPL
WT
pHi
^2
6.71
7.00
6.59
6.92
7.23
6.57
NC_159
PhaF_P
AtpF_20
D
CIP
GPL
WT
pHi
6.32
7.56
6.33
6.80
6.99
6.27
NC_159
PhaF_P
AtpF_20

Supp Fig 10
