# Supplementary material for: Loss of essential outer membrane functions causes drug hypersensitization in Acinetobacter baumannii overexpressing multidrug efflux pumps
Source: mBio. 2026 Jun 24;17(8):e00902-26. doi: 10.1128/mbio.00902-26 (PMC13463702; doi:10.1128/mbio.00902-26)
Supplement: Supplementary file 2 [file mbio.00902-26-s0009.pdf]

Supplementary Data 1 - Strains, Plasmids, and Primers Used.xlsx

Supplementary Data 2 - 12,000 Guide Library Details.xlsx

Supplementary Data 3 - GPS & WT Data, 7-8 Generations or 14-16.xlsx

Supplementary Data 4 - GPS & WT Data, 7-8 Generations or 14-16 Continued.xlsx

Supplementary Data 5 - CIP CRISPRi Screen Notes & CIP vs Untx Mult T Tests.xlsx

Supplementary Data 6 - CIP CRISPRi Screen FQR vs WT Mult T Tests.xlsx

Supplementary Data 7 - CIP CRISPRi Screen Induced vs Uninduced Mult T Tests & Neutral to Defect in CIP.xlsx
